# Roasting or heating increases elicitation capacity of peanut allergens but does not affect their sensitisation potential in a brown Norway rat model for food allergy

**DOI:** 10.1186/2045-7022-1-S1-O20

**Published:** 2011-08-12

**Authors:** Stine Kroghsbo, Neil Rigby, Yvonne Vissers, Clare Mills, Charlotte Madsen

**Affiliations:** 1 DTU National Food Institute, Soborg, Denmark; 2 Institute of Food Research, Norwich, UK; 3 Wageningen University, Wageningen, Denmark

## Background

Allergenic potential of processed food allergens has primarily been studied by their IgE-binding capacity (elicitation). Roasting of peanuts has been shown to increase IgE-binding capacity. In this study we examined whether processing of whole peanuts or of the major peanut allergen Ara h 1 influenced the sensitisation potential.

## Methods

Brown Norway rats were either dosed orally by gavage each day for 42 days with finely ground whole peanut products (blanched or roasted peanuts or peanut butter) mixed with water [~2 mg Ara h 1/rat/day] or immunised i.p. three times with 200 μg of native, heated or heat glycated Ara h 1. Sera obtained at sacrifice were analysed for specific IgG and IgE by ELISA and for biological functionality of IgE by rat basophilic leukaemia (RBL) assay.

## Results

Processing was found to decrease solubility and thus extractability of Ara h 1 from peanut products. Aggregation state and secondary structure changes induced by heating of purified Ara h 1 were identical to those observed when Ara h 1 was heated in the presence of glucose. Although a significant anti-Ara h 1 IgE response was only found when dosing rats with roasted peanuts, examination of functional specific IgE by RBL assay showed that processing of peanuts did not influence sensitisation potential. However, extract from roasted peanuts was found to be a superior elicitor compared to extract from blanched peanuts irrespective of the peanut product used for sensitisation. Processing of purified native Ara h 1 did not influence the sensitisation capacity. Nonetheless, ELISA results indicated that new epitopes are formed or disclosed by heating of Ara h 1. Furthermore, IgG1-binding capacity was found to reflect whether rats were sensitised to native or processed Ara h 1 or dosed with blanched or roasted peanut products.

## Conclusion

Roasted peanuts, either as such or as peanut butter, do not have a higher sensitisation capacity than blanched peanuts. This is supported by the finding that process-modified Ara h 1 has a similar sensitisation capacity as native Ara h 1. Yet, our results show that roasting increases elicitation capacity.

